# Computational studies on visceral artery lesions and aortic pathologies involving visceral branches: a comprehensive review for the clinician

**DOI:** 10.3389/fcvm.2026.1856457

**Published:** 2026-07-10

**Authors:** Siting Li, Tianai Wu, Xiaoning Sun, Xiao Liu, Zenghan Cao, Fangda Li, Rong Zeng, Shiyi Yang, Yuehong Zheng

**Affiliations:** 1 Department of Vascular Surgery, Peking Union Medical College Hospital, Chinese Academy of Medical Sciences & Peking Union Medical College, Beijing, China; 2 Department of State Key Laboratory of Complex Severe and Rare Diseases, Peking Union Medical, College Hospital, Chinese Academy of Medical Science and Peking Union Medical College, Beijing, China; 3 Key Laboratory of Biomechanics and Mechanobiology (Beihang University), Ministry of Education, Beijing Advanced Innovation Center for Biomedical Engineering, School of Biological Science and Medical Engineering, Beihang University, Beijing, China

**Keywords:** aneurysm, biomechanics, computational fluid dynamics, dissection, visceral artery

## Abstract

Visceral arteries, including the celiac artery, superior mesenteric artery, and renal arteries, play a critical role in supplying blood to abdominal organs. Lesions affecting these arteries can be classified into two categories: primary visceral artery pathologies and secondary involvement due to aortic diseases, sometimes leading to serious complications such as ischemia, organ failure, and rupture. Computational modeling approaches have emerged as a valuable tool for analyzing complex hemodynamics in artery diseases. This review provides a critical overview of computational fluid dynamics (CFD) studies on lesions of or involving visceral arteries. CFD could help understanding how biomechanical forces contribute to the development and growth of aneurysms, stenosis, and dissections, as well as predicting the risk of thrombosis, restenosis, and graft failure after endovascular repair of aortic lesions involving the visceral branches. Significant challenges remain, including model simplifications, the lack of patient-specific boundary conditions, and inadequate clinical imaging data due to the complex and individualized anatomy of visceral arteries. Advancements in fluid-structure interaction modeling, real-time hemodynamic data acquisition from imaging modalities, and machine learning-based image segmentation are expected to enhance simulation accuracy and clinical relevance. CFD has the potential to refine the diagnosis and treatment of visceral artery diseases and improve clinical outcomes.

## 1 Introduction

Visceral arteries including the renal artery, celiac artery, and mesenteric artery, are crucial sources of blood supply to all abdominal organs. Acute or chronic stenosis of visceral arteries could lead to extensive visceral ischemia, and subsequentially severe complications such as intestinal necrosis and kidney dysfunction (1, 2). Aneurysms of the visceral arteries are more insidious but can carry a high risk of mortality if ruptured (3). For complex aortic diseases involving the thoracic and abdominal aorta, reconstruction of visceral artery branches remains a challenge in both open and endovascular treatments (4). Treatment of visceral artery stenosis and aneurysms, along with the reconstruction of visceral artery branches, often involves the sacrifice of blood supply to the diseased vessels and/or their secondary branches. Accordingly, evaluation of collateral circulation is crucial for determining the necessity and expected efficacy of interventions for stenotic lesions. Individualized assessment is also essential for estimating the risk of ischemia after aneurysm embolization and for selecting appropriate strategies for visceral artery reconstruction. However, an efficient and quantitative tool to evaluate collateral capacity and predict hemodynamic consequences is still lacking.

Hemodynamic conditions play a significant role in arterial stenosis and dilation (5). However, due to the complex anatomy of visceral arteries, there is currently a lack of quantitative evaluation tools for these vessels, as well as limited understanding of the causes of aneurysms and the risk factors for long-term stenosis of visceral branches. Computational fluid dynamics (CFD) offers a convenient, non-invasive, and accurate means to assess hemodynamic status in blood vessels (6). It has been extensively applied to the evaluation cardiovascular diseases such as the coronary artery, intra-cranial aneurysm, and aortic diseases (7, 8). In recent years, with advancements in imaging and computational techniques, CFD is increasingly being used to assess visceral artery lesions and complex aortic diseases that involve reconstruction of visceral arteries (9, 10).

This review is designed particularly for clinicians who may not be familiar with CFD methodologies. This review aims to provide an accessible overview of the fundamental steps involved in CFD simulation, the clinical imaging and physiological data required to construct accurate models, and the key hemodynamic variables that CFD can quantify. Additionally, this review summarizes computational studies in this field, explicitly distinguishing between primary visceral artery pathologies and secondary involvement from aortic disease. Selected representative studies are outlined in Tables 1, 2, examining their applications across disease progression assessment, surgical planning, and complication prediction. Finally, the review discusses the limitations and challenges of CFD modeling and outlines future directions in visceral artery research.

**Table 1:** Selected studies on visceral artery lesions.

Author(s)	Main design	Model numbers	Boundary conditions and images	Key findings
Mesenteric artery				
Park et al. (21)	CFD study of SISMAD	One ideal model with different branch angles	Pressure boundary, ideal model	Abnormal stresses were observed at the anterior wall around the convex portion of the SMA in SISMAD patient.
Jia et al. (23)	FSI study of SMAD	One patient model with different angle and curvature	Velocity inlet and pressure outlets, CTA	Increased aortomesenteric angle and SMA curvature were associated with higher and more concentrated WSS and thus SMAD development.
Mei et al. (24)	CFD study of SMAS vs. SMAD	30 SMAS and 30 SMAD patient models	Patient-specific velocity inlet, CTA	Higher turbulent kinetic energy (TKE) and lower blood flow velocity (BFV) at the root of SMA were observed for SMAS, while lower WSS in the curved segment was observed for SMAD.
Zhang et al. (26)	CFD study of ISMAD classification	62 SISMAD patient models	Velocity inlet and pressure outlets from reference, CTA	Endovascular treatment was recommended for type II SISMAD that had significantly low TAWSS and high OSI, as well as for type IV stenotic and type V SISMAD.
Wei et al. (29)	CFD study of SISMAD	One patient model	Velocity inlet from literature and pressure outlets, CTA	TL exhibits high velocity, high WSS near the entry tear. FL shows low flow, stasis, low WSS, and high OSI.
Xu et al. (28)	CFD study of a new SISMAD classification	70 SISMAD patient models	Constant velocity inlet and outlet, CTA	A new HX classification based on false lumen (FL) flow is proposed: Type I (flow in FL) and Type II (thrombosed FL).
Pancreaticoduodenal artery				
Yuhn et al. (33)	CFD study of PDAAs with CA stenosis	8 PDAA patient models including 5 normal and 3 with celiac artery stenosis	1D–0D model with iterative arterial diameter change until TAWSS reached homeostatic, verified by CTA	Remodeling of the PDA resulted in increased diameter and blood flow redistribution to maintain adequate organ perfusion regardless of stenosis severity.
Li et al. (32)	CFD study of VAAs including PDAAs	13 VAA patient models	Pressure and velocity inlets from references, CTA	Both local increase and decrease of WSS and WSS gradient were observed for the VAA forming area compared to para-aneurysm area.
Celiac trunk and splenic artery				
Tatari et al. (34)	CFD study of distal and proximal splenic artery embolization	One patient model with or without collaterals and coil placement	Velocity inlet and resistance outlets from reference, CTA	Collateral vessels helped maintain spleen perfusion after proximal embolization, and proximal embolization achieved higher efficiency in reducing pressure while preserving collateral flow.
Gao et al. (35)	CFD study of VAAs	6 patient models with different time-phase geometries	Velocity inlet and pressure outlets from references and adjusted to patient's ECG-gated map, 4D-CTA	Periodic deformation and displacement of VAAs had a dominant but generally minor influence on hemodynamics​, and WSS had a larger coefficient of variation than velocity and pressure.
Hepatic artery				
Childress et al. (38)	FSI study of hepatic artery system	One ideal hepatic artery system model in flexible and rigid cases	Velocity inlet and pressure outlets from reference, enhanced CT	The time-averaged rigid geometry provided the best approximation with the flexible arterial walls for catheter placement of direct tumor-targeting during the diastolic phase of the cardiac cycle.
Du et al. (40)	CFD study of HA in early biliary atresia with hepatic fibrosis	40 HA patient models	velocity inlet and resistance outlets from reference, enhanced CT	Hemodynamic parameters (flow distribution ratio, pressure, WSS, and energy loss) in hepatic arteries increased with the progression of hepatic fibrosis.
Renal artery				
Kagadis et al. (41)	CFD study of RAS	One patient model in the healthy, stenotic, and post-stent states	Velocity inlet and pressure outlets from reference, enhanced CT	Severe RAS significantly increased pressure gradients and flow resistance, while stent implantation restored near-normal hemodynamics.
Zhao et al. (47)	CFD study of RAS	One patient model with different degree and position of stenosis	Velocity inlet and pressure outlets from references, CTA	Renal perfusion decreased with increasing stenosis severity and distance from the aorta.
Csonka et al. (51)	CFD study of RBA	One idea model with different RBAs	Velocity inlet and pressure outlets from references, CTA	An optimal RBA range was found with relative constant pressure, volume flow, and velocity for renal transplant patients
Soliveri et al. (48)	CFD study of RAS	6 FMD patients, 2 atherosclerotic RAS patients, 2 healthy volunteers	Patient-specific peak-systolic blood flow at inlet, calibrated resistances at outlets, CTA/MRI	Computed fractional flow reserve and pressure drops.

CFD, computational fluid dynamics; FSI, fluid-structure interaction; SISMAD, spontaneous isolated SMA dissection; SMAS, superior mesenteric atherosclerotic stenosis; PDAA, pancreaticoduodenal artery aneurysm; VAA, visceral artery aneurysm; HA, hepatic artery; RAS, renal artery stenosis; RBA, renal branch angle.

**Table 2:** Selected studies on aortic lesions involving visceral branches.

Author(s)	Main design	Model numbers	Boundary conditions and images	key findings
Aortic aneurysm				
Šutalo et al. (53)	CFD study of antegrade vs. retrograde flow stent	3 branch geometries	Pulsatile flow inlet, pressure outlet based on experiments, ideal models	For a long conduit, retrograde flow resulted in a lower outflow compared to antegrade.
Kandail et al. (54)	CFD study of BSGs vs. FSGs	8 BSGs and 8 FSGs with different visceral ToA and lateral aortic neck angles	Velocity inlet and pressure outlets from references, ideal models	Blood flow rate in renal arteries depends on the configuration of the stent-graft, with an FSG giving maximum renal flow and a retrograde BSG resulting in minimum renal flow.
Kandail et al. (55)	CFD study of flared vs. non-flared renal stents in FEVAR	1 non-flared, 5 flared FSGs	Velocity inlet and 3-element Windkessel outlets, ideal models	Flaring maintains sufficient renal perfusion and does not alter renal flow waveforms,but it creates flow disturbance and high ECAP
Suess et al. (56)	CFD study of 4 stent graft configurations	4 stent graft configurations	Velocity inlet and pressure outlets from references, ideal models	Abrupt 90°and 180° changes in stent geometry cause a high momentum change and increased flow separation and mixing in renal branch. Longer bridging stents provide more gradual changes in momentum.
Georgakarakos et al. (58)	FSI study of the pivotal fenestrated endograft	5 angulations of the left renal branch	Velocity inlet and pressure outlets from references, ideal models	The proximal part of the renal branch preserved constant WSS. The variant horizontal branch orientation influences the WSS distribution across its length and affects its values at its transition with the mating vessel.
Ou et al. (59)	CFD study of fEVAR	5 custom-made vs. 5 p-branch models	Velocity inlet and pressure outlets from references, ideal models	Custom-made models and those navigating the renal stents towards caudal orientation exhibited higher flow rate and WSS and smaller FRZ in renal arteries.
Tricarico et al. (60)	CFD study of chEVAR	6 chEVAR.	Velocity inlet and pressure outlets from references, CTA	Chimney stent-grafts at increased risk for occlusion demonstrated anatomic and hemodynamic signatures (lumen area, systolic pressure gradient and systolic WSS) within 1 month of juxtarenal chEVAR.
Moulakakis et al. (62)	CFD study of fEVAR vs. ChEVAR	1 FEVAR vs. 1 ChEVAR	Velocity inlet and pressure outlets from references, CTA	Reduction in perfusion in both renal arteries was observed in both fEVAR and ChEVAR. Lower decrease in fEVAR.
Tran et al. (10)	CFD study of fEVAR	10 preoperative models vs. 10 postoperative models	Velocity inlet and pressure outlets from references adjusted to patient's characteristics CTA	Structural changes in aortic flow geometry after fEVAR did not adversely impact estimated renal or visceral branch perfusion metrics or WSS
Tran et al. (63)	CFD study of fEVAR vs. bEVAR	10 fEVAR vs. 10 bEVAR	Velocity inlet and pressure outlets from references adjusted to patient's characteristics, CTA	bEVAR is associated with subtle decreases in renal perfusion and a large increase in WSS pared with fEVAR. No significant differences between celiac artery or SMA perfusion.
Malatos et al. (65)	CFD study of fEVAR vs. ChEVAR	3 FEVAR vs. 3 ChEVAR	Velocity inlet and pressure outlets from references, CTA	Both modalities reduced the FRZ in the main graft and aortic branches. ChEVAR presented lower FRZ parallel grafts' entries while fEVAR showed less intense flow regurgitation in visceral stents.
Brand et al. (61)	CFD study of ChEVAR	2 ChEVAR	Velocity inlet and pressure outlets from references, ideal models	The short chimney is hemodynamically viable and may reduce procedural time.
Wang et al. (64)	CFD study of fEVAR/bEVAR	6 fEVAR/bEVAR	Velocity inlet and pressure outlets from references, CT	A larger tilt angle of the branch stent, smaller branch entry depth, and larger branch stent diameter improve blood flow
Aortic dissection				
Jiang et al. (66)	CFD study of PMEG	6 AD before and after the PMEG	Velocity inlet and pressure outlets from references, CTA	PMEG can restore the abnormal blood supply of the visceral arteries according to flow rate, pressure, TAWSS, RRT and ECAP.
Lee et al. (67)	FSI study of AD by AC and AFC	1 AC and 1 AFC	Velocity inlet and pressure outlets from previous 4D flow MRI experiments in an *in vitro* model, ideal models	The pressure difference between the true lumen and false lumen allows the intimal flap to block blood flow to the CA and SMA in AD model when using AC as the cannulation flow rate increased. The amount of visceral perfusion of AFC was higher than that of AC.
Kimura et al. 2024 (68)	CFD study of AAD	1 TEVAR	Velocity inlet and pressure outlets from references, CTA	FL depressurization achieved by TEVAR resulted in TL expansion and increased blood flow to the visceral vessels. TEVAR increased perfusion volume of the visceral vessels.
Other aortic lesions involving visceral branches				
Scardulla et al. (70)	CFD study of LVAD	5 LVAD	Boundary conditions according to echocardiographic measurements and a 3D-printed anatomic model experiment, CTA	Highest WSS was observed in the left gastric artery and a positive correlation was found between celiac trunk angulation and WSS distal to the ostium, suggesting a potential mechanism for LVAD-associated gastrointestinal bleeding.
Tossas-Betancourt et al. (71)	FSI study of abdominal aortic coarctation	1 SAAC preoperative model, 3 TABs, 3PAs, 1 healthy model	Calibrated boundary condition acquired from PC-MRI, CTA	Both TAB and PA improved renal artery flow and pressure waveforms. Only the 0% PA oversizing scenario eliminated all high frequency disturbances.

CFD, computational fluid dynamics; BGS, branched stent-grafts; FGS fenestrated stent-grafts; ToA, takeoff angles; FRZ, flow recirculation zones; fEVAR, fenestrated endovascular aneurysm repair; chEVAR, chimney endovascular aneurysm repair; bEVAR, branched endovascular aneurysm repair; FSI, fluid-structure interaction; PMEG, patients with aortic dissection underwent; AAD, acute aortic dissection; TEVAR, thoracic endovascular aortic repair; LVAD, left ventricular assist device; SAAC, suprarenal abdominal aortic coarctations; TAB, thoracoabdominal bypass; P.

## 2 Methods

This review was conducted in accordance with the Preferred Reporting Items for Systematic Reviews and Meta-Analyses (PRISMA) 2020 statement (11). PubMed/MEDLINE, Scopus, Web of Science, and Embase were searched for articles published between January 1, 2006, and December 1, 2025. The reference lists of all included articles were manually screened to identify additional relevant studies. No language or date restrictions were applied.

The search strategy was constructed using three concepts combined with the Boolean operator “AND”: (1) computational modeling and biomechanics methods, (2) anatomical location of visceral arteries and the associated aorta, and (3) relevant pathologies or endovascular treatment techniques. Search terms are presented in Appendix A.

The inclusion criteria were designed to capture studies with direct clinical relevance to specific visceral artery pathologies. Studies were included if they: (1) investigated a named clinical disease entity affecting the visceral arteries or aortic pathologies involving visceral branches; (2) performed computational fluid dynamics (CFD) or fluid-structure interaction (FSI) simulations; and (3) reported hemodynamic parameters with potential clinical implications, such as wall shear stress (WSS) and its derivatives, oscillatory shear index (OSI), pressure gradients, flow distribution, or energy loss. Although the primary focus of this review is CFD, we also included studies employing FSI because it incorporates CFD as its fluid solver, providing additional insights into wall compliance and are increasingly used in visceral artery research. FSI studies are clearly identified as such in Tables 1, 2.

Studies were excluded if they: (1) were review articles, case reports without computational analysis, conference abstracts, editorials, and letters; (2) were purely *in vitro* or animal studies lacking a computational modeling component; (3) focused on vascular territories other than the visceral arteries; or (4) unavailable for full text.

**Figure 1:**
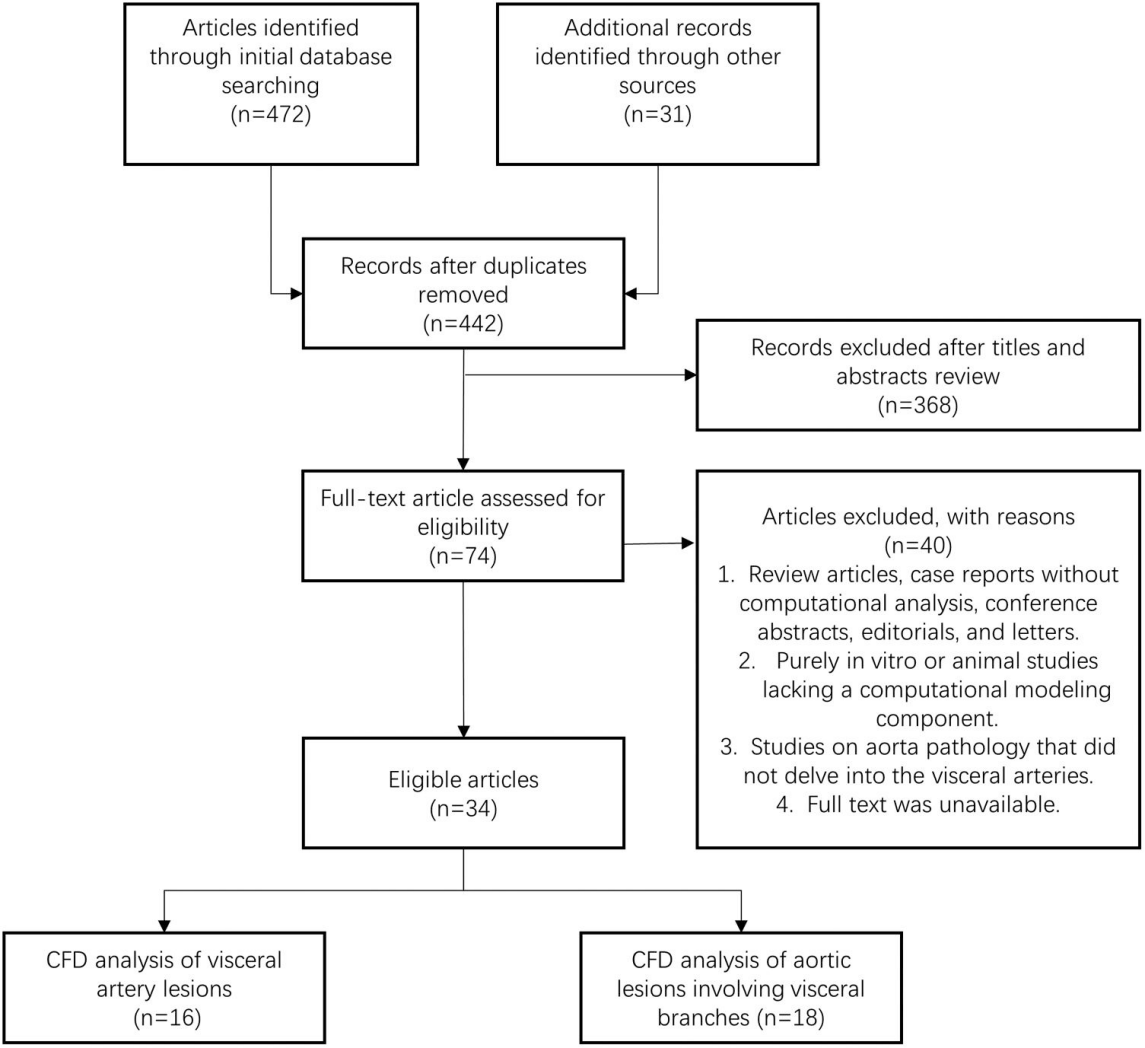
Search strategy and selection criteria.

All retrieved records were imported into EndNote (Clarivate Analytics, PA, USA) for duplicate removal and management. Two authors independently screened the titles and abstracts of the remaining records to identify potentially relevant studies. The full texts of these selected articles were then retrieved and assessed for final eligibility. Studies were included if they employed computational modeling techniques such as CFD and FSI for biomechanical analysis and focused on lesions of the visceral arteries or aortic lesions involving the visceral branches. Review articles, case reports without computational analysis, conference abstracts, editorials, and letters were excluded. The flow chart depicting the process for search strategy and selection criteria are presented in Figure 1.

A quality checklist specific to all eligible studies was used to assess all included studies (Supplementary Table S1). Two authors independently scored each study; disagreements were resolved by consensus. Individual study scores are provided in Supplementary Table S2.

## 3 Computational fluid dynamics: basis and steps

For many clinicians, CFD may be an unfamiliar methodology. This section provides a concise, practical overview of how CFD models are built, what clinical data they require, and what types of information they can—and cannot—reliably produce. By outlining the basic workflow, this overview aims to help clinicians better understand the strengths and limitations of CFD, as well as its appropriate role in assessing visceral arterial disease. As summarized in Table 3, these tools can be categorized into four key stages: image segmentation, mesh generation, numerical simulation, and post-processing, each contributing uniquely to the translational pathway. The selection of appropriate software platforms at each stage of the CFD workflow is not merely a technical detail—it directly influences the feasibility, reproducibility, and ultimately the clinical applicability of hemodynamic simulations.

**Table 3:** Computational simulation platforms.

Software	Area	Characteristics
Mimics	Pre-processing	Software suite used for medical image processing and 3D modeling
3D Slicer	Pre-processing	Open-source software platform for medical image visualization, analysis, and processing
ITK	Pre-processing	Open-source software system for image analysis, processing, and registration
VTK	Pre-processing	Open-source software system for 3D computer graphics, image processing, and visualization
Matlab/Python	All areas	Computing environment providing tools for data analysis, algorithm development, and visualization
Gambit	Mesh generation	Specifically designed for CFD simulations supporting various mesh types
Meshmixer	Mesh generation	Free computer-aided design (CAD) software providing powerful tools for creating and editing 3D meshes
TGrid	Mesh generation	Unstructured grid generation program designed for CFD simulations
ICEM CFD	Mesh generation	Specifically designed for CFD simulations supporting various meshing techniques
ANSYS fluent	Numeric simulation	Widely used for simulating fluid flow, heat transfer, and chemical reactions using FVM methods
		Widely used for general fluid flow, including large-vessel hemodynamics; less specialized for cardiovascular boundary conditions but robust and well-documented
Tecplot	Post-processing	Visualization and analysis software supporting a wide range of formats for importing data
Paraview	Post-processing	Open-source visualization and analysis software supporting a wide range of data formats
FEniCS	Solver	Open-source platform for automated solution of differential equations using the Finite Element Method.
SU2		Open-source CFD solver with adjoint-based optimization capabilities, suited for aerodynamic and vascular shape optimization.
Nektar++		Open-source spectral/hp element method solver for high-fidelity simulations.
HemeLB		Open-source solver based on Lattice Boltzmann methods, designed for large-scale blood flow simulation.
ANSYS workbench	Integrated software	Platform incorporating ANSYS's suite of simulation tools with user-friendly environment. Suitable for general vascular CFD but less specialized for cardiovascular boundary conditions.
SimVascular	Integrated software	Open-source software for cardiovascular simulation using FEM methods, supporting for patient-specific boundary conditions and FSI.
CRIMSON	Integrated software	Open-source software for cardiovascular simulation using FEM methods, optimized for research use in complex arterial geometries.
COMSOL	Integrated software	Multiphysics simulation software using FEM methods
XFlow	Integrated software	Particle-based Lattice Boltzmann technology solver
OpenFOAM	Integrated software	Open-source CFD package; highly customizable, FVM-based, suited for complex fluid dynamics but requires programming expertise

CFD, computational fluid dynamics; FVM, finite volume method; FEM, finite element method.

The science governing the physics of blood flow is termed hemodynamics. Fundamentally, blood can be modeled as obeying the Navier–Stokes equation while CFD approaches vary in complexity. Low-dimensional simulations (0D or 1D) are computationally efficient but simplify the vascular system into lumped or tubular segments, which limits their ability to represent the complex geometry and flow patterns seen in individual patients (12). High-dimensional (2D or 3D) simulations provide much more detailed hemodynamic information but require accurate anatomical models and substantially more computational resources. An important consideration often overlooked in clinical CFD studies is the choice of flow regime assumption. Visceral artery stenoses and chimney EVAR (chEVAR) configurations induce highly complex, transitional, or turbulent flow structures rather than strictly laminar. Laminar flow assumptions, while computationally convenient, may underestimate energy loss and misrepresent wall shear stress distribution in these regions. Reynolds-Averaged Navier-Stokes (RANS) models are computationally efficient and provide time-averaged flow fields. However, they cannot resolve instantaneous flow fluctuations and may fail to capture complex vortex shedding or FRZs, which are critical for predicting thrombus formation or wall remodeling. Large Eddy Simulation (LES) models smaller scales while analyzing larger turbulent eddies, providing higher fidelity for unsteady 3D flow structures. It is particularly valuable in geometries with abrupt caliber changes, such as the transition from aorta to small visceral branches or at fenestration edges.

The first requirement for 3D CFD is a patient-specific vascular geometry. Typically, patients with vascular diseases undergo computed tomography angiography (CTA) or magnetic resonance angiography (MRA) to evaluate their lesions. From these imaging modalities, a 3D vascular model can be segmented by delineating vessel regions and separating them from surrounding organs on platforms such as Mimics (commercial) and 3D Slicer (open-source). Subsequently, the region of interest should be isolated from the vessel system. The geometric model can then be smoothed in Computer-Aided Design (CAD) software to facilitate subdivision into nodes and elements. Notably, the inlet and outlet of vessels should be trimmed with flat surfaces to enable the addition of boundary conditions (BCs). While this step is necessary for computation, it already introduces deviation from the true clinical anatomy.

Boundary conditions are a critical—and often the most challenging—component of clinically meaningful CFD (13). Velocity flow at the vessel inlet or outlet could be obtained from doppler ultrasound (DUS). However, the hemodynamic data is often incomplete or inaccurate due to poor acoustic windows, deep vessel locations, or operator dependency. Two-dimensional cine phase contrast-MRI (2D cine PC-MRI) is another non-invasive technique providing direct and accurate measurements of flow velocity and volume. But it is also limited to obtaining velocity components within a single two-dimensional plane. Three-dimensional (3D) PC-MRI captures the full 3D velocity vector in all directions, providing hemodynamic data over the whole scanning area. Four-dimensional (4D) PC-MRI can provide instantaneous 3D velocity information at each point of the vessel model. However, the time-consuming and costly nature of MRI may limit its availability. For patients undergoing CTA, time-varying 1D velocity can be extracted from the blood flow spectrum curve of Doppler ultrasound. Besides velocity, invasive pressure recordings can be used but are seldom available for all relevant branches. Physiologic factors such as respiration, variable cardiac output, and post-prandial changes further complicate the interpretation of flow data. Outflow waveforms are particularly difficult to measure because visceral arteries terminate in organ beds with complex, dynamic microcirculation. As a result, many studies use mathematical models, most commonly the three-element Windkessel model (14, 15), to approximate the resistance and capacitance of proximal vessels (arteries) and the resistance of distal vessels (arterioles and capillaries). This lumped parameter approach provides a computationally efficient means of representing global flow distribution. However, it relies on simplified assumptions about distal vascular resistance and therefore cannot fully capture the dynamic behavior of complex collateral networks or organ specific autoregulation. More advanced impedance boundary conditions, such as structured-tree outflow models, can mitigate artificial wave reflections at truncated outlets and better represent the frequency-dependent behavior of the distal vascular bed.

With patient-specific geometric models and their associated boundary conditions, we can simulate hemodynamics by solving these equations using computational methods. Firstly, the geometric models should be spatially partitioned into small mesh grids to enable the solution of the differential equation at discrete points. Mesh quality significantly impacts simulation reliability, although overly refined meshes may escalate computational burden. Generally, tetrahedral or polyhedral mesh are used for inside blood lumen, while triangular or prismatic surface mesh are used for the boundary wall. Mesh generation can be facilitated using specialized tools (e.g., Gambit) or integrated software (e.g., Ansys), which are outlined in Table 3.

Simulation methods encompass the finite element method (FEM), finite difference method (FDM), finite volume method (FVM), boundary element method (BEM), and finite analytic method (FAM). FVM-based solvers (e.g., Ansys Fluent) are widely used for their robustness in large-vessel flow simulations, while FEM-based platforms (e.g., CRIMSON) are better suited for FSI analyses where wall compliance is of interest.

Despite employing distinct numerical technique, the basis of the solver is to perform an approximation of unknown flow variables (6). Standard CFD generally assumes rigid vessel walls. However, real arteries deform with pulsatile pressure, disease progression, and stent implantation. Fluid–structure interaction (FSI) modeling attempts to account for this by coupling blood flow with vessel wall deformation. FSI allows simulation of wall motion, stent–artery interaction, and the dynamic changes of dissected lumens. Nevertheless, its accuracy is limited because vessel wall elastic properties, anisotropy, and regional weakening are rarely known for individual patients.

The hemodynamic results are calculated for derived flow variables and visualized through post-processing (Table 3). The flow-induced variables could be associated with disease progress in patient-specific models. The output is visualized using streamlines, pressure maps, wall shear stress (WSS) contours, and particle-tracking simulations, which clinicians can interpret more easily. Certain commercial or open-source software have integrated these methods, rendering them user-friendly for clinicians. These integrated software incorporate the entire computational simulation process, encompassing geometry smoothing, mesh generation, fluid property setting, equation solving, thrombosis modeling, and post-processing, including parameter calculation and visualization. The characteristics and source of some of these commonly used software are listed in Table 3.

Simulations offer a powerful tool for quantitatively assessing key hemodynamic parameters within visceral arterial systems. Among these, Wall Shear Stress (WSS) and its derived metrics, such as the Oscillatory Shear Index (OSI) and Relative Residence Time (RRT), are crucial for understanding endothelial mechanotransduction and the initiation and progression of vascular pathologies. Furthermore, CFD provides detailed insights into pressure distributions, enabling the evaluation of pressure gradients across stenotic lesions or within aneurysms, which directly correlate with perfusion deficits or rupture risk. Velocity profiles and complex flow patterns, including recirculation zones and secondary flows, can also be precisely characterized, elucidating areas of disturbed flow that contribute to atherogenesis, thrombosis, and altered nutrient exchange. The trans-lesional pressure gradient, defined as the systolic or mean pressure drop across a stenotic lesion, directly reflects the hemodynamic significance of a stenosis. Fractional flow reserve (FFR), the ratio of distal to proximal pressure under maximal hyperemia, has become the gold standard for guiding revascularization in the coronary circulation. This concept has been extended to renal and mesenteric arteries using CFD. Energy loss (EL) represents the dissipation of mechanical energy due to viscous friction, flow separation, and vortices. It is calculated as the difference in total pressure between the inlet and outlet of a vascular segment, often normalized by flow rate or time-averaged. Elevated EL indicates inefficient flow and has been associated with aneurysm growth, thrombus formation, and post-stent remodeling. Unlike WSS, which is a local wall metric, EL provides a global assessment of flow efficiency within a vascular region. Particle tracking simulations offer invaluable insights into fluid pathways, residence times of blood components, and the potential for thrombus formation or embolic release, particularly in regions of disturbed flow. Collectively, these comprehensive hemodynamic data derived from CFD are indispensable for unraveling the intricate mechano-biological mechanisms underlying the diseases. Nevertheless, CFD cannot fully simulate biological processes such as endothelial remodeling, vessel wall inflammation, or organ-specific autoregulation. It provides a mechanical perspective, not a complete physiological one. Careful validation against real clinical data, such as comparisons with invasive pressure measurements, Doppler or MRI flow data, or postoperative imaging outcomes, are needed to verify whether simulations accurately reflect physiologic conditions. Such validation also helps refine boundary conditions and improve model performance.

In summary, CFD provides clinically valuable insight into hemodynamic mechanisms of visceral artery disease, but its accuracy depends heavily on image quality, boundary conditions, and proper validation. Understanding these strengths and limitations is essential for clinicians interpreting CFD results or considering their integration into clinical decision-making.

## 4 CFD analysis of visceral artery lesions

CFD has become an increasingly useful tool for exploring the hemodynamic mechanisms underlying visceral artery lesions. For clinicians who may be less familiar with computational modeling, this section summarizes the major CFD findings related to the superior mesenteric artery, celiac–mesenteric collateral pathways, celiac trunk branches, and renal arteries. Emphasis is placed on how CFD contributes to understanding disease initiation and progression, and on the practical challenges that arise when simulating visceral arteries, particularly the difficulty of defining accurate boundary conditions in vessels with rich collateral networks and physiologically variable flow patterns.

### 4.1 The superior mesenteric artery

The superior and inferior mesenteric arteries are critical for intestinal perfusion. The superior mesenteric artery (SMA) supplies the midgut, while the inferior mesenteric artery (IMA) supplies the hindgut, including the descending colon, sigmoid colon, and rectum. In our systematic search, we used the broad keyword “mesenteric” to capture studies on both mesenteric arteries. However, no CFD or FSI studies specifically addressing the IMA met our inclusion criteria. By contrast, numerous studies on the SMA were identified.

The lack of IMA-specific CFD research may be attributed to its relatively small caliber and tortuous course, which make image segmentation and boundary condition assignment more challenging. And the historical perception that the IMA is less hemodynamically critical than the SMA or renal arteries in most visceral pathologies. This absence encourages future studies to include the IMA, particularly in the context of EVAR and collateral circulation assessment.

Much CFD research on visceral arteries has focused on studying the hemodynamic conditions of the SMA in both physiological and pathological states. Acute embolic/thrombotic occlusion or dissection of the SMA can cause life-threatening ischemia and require emergent surgery. Chronic SMA stenosis is also associated with significant clinical symptoms that may necessitate intervention (16).

Jeays and colleagues pioneered the investigation of SMA hemodynamics by developing a patient-specific, time-dependent aorta-SMA segment model (17). Their analysis of time-averaged wall shear stress (TAWSS) and oscillatory shear index (OSI) revealed comparable results between rigid and motion-walled SMA models throughout the cardiac cycle. Notably, they identified a correlation between elevated OSI and atheroma formation in regions proximal to the bifurcation. The team further validated their findings by comparing lattice Boltzmann and finite element methodologies (18). Mabotuwana and colleagues developed a one-dimensional CFD model incorporating all major mesenteric arteries (19). Their calculations of pressure and velocity distributions during key cardiac phases—diastole, end-isovolumic contraction, peak ejection, and peak isovolumic relaxation—demonstrated strong alignment with physiologically realistic profiles. Sugiyama et al. explored the flow in SMA under normal and post-prandial states in models of seven healthy volunteers, and found that the most significant flow changes occurred at the distal straight section of the SMA, as measured by 2D cine phase-contrast (PC)-MRI (20).

Beyond physiological studies, researchers have extensively explored computational analyses of pathological SMA conditions. Park and colleagues conducted a comparative analysis of spontaneous isolated SMA dissection (SISMAD) cases and combined aortic-SMA dissections at their institution (21). Similar to clinical results, CFD analysis revealed abnormal mechanical stresses on the anterior wall of the SMA, particularly in the transition zone from a fixed to a relatively unfixed portion. In a subsequent study, Wu et al. measured the models of SISMAD and normal controls and found that SISMAD patients had a larger mean SMA-distal aorta angle (22). FSI studies indicated higher stress in the arterial wall and elevated OSI levels at the SMA's superior convexity, which increased with the angle. Jia et al. also conducted FSI simulation of SMAD and identified aortomesenteric angle and SMA curvature as risk factors for SMAD development (23). Histology analysis of SMA specimen showed thinner thickness of media and lower fractions of elastin and collagen in the anterior wall of the SMA curve. Their team further explored hemodynamic factors contributing to superior mesenteric atherosclerotic stenosis (SMAS) and SMAD (24). Higher turbulent kinetic energy (TKE) and lower blood flow velocity (BFV) at the root of SMA were observed for SMAS compared to SMAD, which was consistent with plaque location, whereas lower WSS in the curved segment was observed for SMAD. The researchers hypothesized that deploying the proximal end of a stent near or into the aortic arch could improve hemodynamic conditions and reduce the risk of in-stent restenosis (25).

Despite general agreement on the role of geometry, discrepancies exist regarding the predictive value of specific metrics. Zhang et al. evaluated the clinical utility of SISMAD classification through hemodynamic parameter analysis (26). The results suggested that endovascular treatment for type II SISMAD (based on a recently proposed classification system) had significantly low TAWSS and high OSI, as well as for type IV stenotic and type V SISMAD (27). A new classification of SISMAD was proposed (HX classification) based on the different blood flow conditions, which recommend the ratio of rupture area to entrance area as a risk factor for Type I (dual-lumen flow type), while the ratio of minimum diameter to maximum diameter for Type II (single-lumen flow type) (28). Wei et al. further demonstrated distinct hemodynamics between the true lumen and the false lumen in Yun Type I SISMAD. The FL showed significantly lower velocities, regions of flow stasis, and low WSS, confirming the pro-thrombotic environment (29). These comprehensive studies underscore the critical role of personalized CFD analysis in guiding therapeutic strategies for SMA-related pathologies.

### 4.2 The celiac-mesenteric anastomosis

Beyond its intestinal supply function, the SMA branches into the pancreaticoduodenal arteries (PDA), forming anastomoses with celiac trunk branches. Some studies hypothesized that PDA aneurysms were associated with hemodynamic alterations in the anastomosis, caused by altered blood flow due to celiac trunk or SMA stenosis or occlusion (30). Several studies have utilized CFD methods to illuminate the underlying mechanisms and potential therapeutic strategies for these lesions.

Mano and colleagues assessed the hemodynamic parameters in five patients using four-dimensional flow-sensitive magnetic resonance imaging (4D-Flow) (31). Multiple regions with extremely high OSI values and higher flow volume in gastroduodenal artery (GDA) and SMA were observed for patients with PDA aneurysm compared to control participants. Li et al. conducted CFD analysis in 14 visceral artery aneurysm (VAA) (aneurysms were on the celiac trunk, SMA, or PDA) models and found both local increase and decrease of WSS and WSS gradient at the VAA forming area (32). Yuhn and colleagues used a one-dimensional arterial model and lumped Windkessel peripheral circulation models to simulate the hemodynamic changes following celiac trunk stenosis (33). The researchers assumed the restoration of normal TAWSS value as the goal of vessel remodeling, and found that adequate blood supply to the organs could be maintained after the adaptation. In a focused case study, Li et al. performed detailed CFD analysis on a three-dimensional model of a PDA aneurysm patient (9). Virtual surgery of the model suggested that compared to aneurysm resection and anastomosis, aneurysmectomy without revascularization could avoid abnormal WSS at the original aneurysm site, and the latter surgical option was selected for the patient. These collective findings demonstrate the valuable role of CFD analysis in understanding hemodynamic alterations associated with PDA aneurysms and optimizing surgical strategies.

### 4.3 The celiac trunk and branches

The celiac trunk divides principally into the left gastric, common hepatic, and splenic arteries. Its anastomotic connection with the SMA typically prevents organ ischemia during trunk stenosis or occlusion (33). Nevertheless, CFD analyses of these arterial branches provide crucial insights into lesion evaluation and blood supply dynamics. Tatari and colleagues developed a three-dimensional splenic artery model to evaluate embolization strategies using the Lagrangian particle tracking method (34). Their results highlighted the critical influence of particle characteristics, deployment location, and timing on embolization success, demonstrating CFD's utility in procedure optimization. Gao et al. explored the effect of VAA's cardiac morphological variation on hemodynamic conditions using 4-D CTA, and concluded that the periodic deformation and displacement on simulation results was insignificant (35).

Computational modeling has also been applied to the hepatic artery system in evaluating hemodynamic changes during liver tumor treatment. Basiano and colleagues simulated the particle release process in a 3D hepatic artery to optimize time and position of microsphere delivery (36), which was validated using an *in vitro* experimental model (37). Their subsequent FSI analyses revealed that diastolic targeting intervals provided optimal conditions for predicting blood and particle transport dynamics throughout the cardiac cycle (38). A detailed summary of CFD modeling in liver radioembolization has been reported (39). Additionally, Du et al. utilized CFD to explore hemodynamic changes in liver fibrosis in patients with biliary atresia and explained why BA patients with a worse degree of hepatic fibrosis always had bad outcomes (40). Compared to the control group, liver fibrosis was associated with an increased hepatic blood flow distribution ratio (FDR) and EL. The results showed that the greater the impact on HA hemodynamics caused by the liver fibrosis, the more energy loss. These investigations collectively demonstrate CFD's instrumental role in advancing treatment strategies for hepatic and splenic artery pathologies while enhancing our understanding of liver fibrosis hemodynamics.

### 4.4 The renal arteries

Most computational studies of the renal artery are focused on renal artery stenosis (RAS), which could lead to renovascular hypertension and renal malperfusion. Kagadis et al. investigated the WSS and differential pressure profiles on the atherosclerotic plaque of a RAS 3D model using a shear stress transport turbulent model, comparing the results to those of healthy and stent-implanted vessels (41). The effects of angulation on blood flow velocity and renal mass flow rate in RAS have also been studied (42). Large renal branch angles can reduce kidney perfusion and consequently activate the renin-angiotensin system, leading to hypertension. Zhang and colleagues built patient-specific CFD models based on MR angiography (MRA) (43). They found that both pressure difference and velocity in the renal arteries increased with the severity of stenosis, while the mass flow rate decreased. Doltra et al. summarized renal artery images from patients who had undergone renal denervation and performed CFD analysis on some of the arteries (44). They observed significant decreases in WSS and increases in mean flow at the 6-month follow-up. Mandaltsi et al. conducted CFD studies on 10 RAS patients, with good consistency between the calculated trans-lesional ratio of distal to proximal pressure and invasively measured results (45). Xiong and colleagues explored the impact of RAS morphology on surrounding hemodynamic conditions (46). The stenotic length, asymmetry, and direction could all influence the WSS and velocity profiles. Zhao and colleagues later pointed out that renal perfusion decreases with an increasing degree of stenosis and more distal stenosis location (47). Soliveri et al. revealed the potential of FFR and trans-stenotic pressure drop to guide revascularization decisions and procedures in patients with RAS (48). FFR in patients ranged depending on the severity and type of the lesion, where there was a perfect correlation between the FFR values derived from the CFD model and the blood flow velocities measured by Doppler ultrasound. These findings validated the utility of CFD in replicating invasive hemodynamic assessments and underscored the potential for non-invasive imaging to replace traditional catheter-based methods in determining the functional severity of RAS.

Stenosis of transplant renal artery is another critical issue. Wang et al. reported the hemodynamic alteration in transplant renal artery (TRA) anastomosed to the external iliac artery (49). Compared to normal TRA, nonuniform pressure distribution, higher maximal velocity, and maximal WSS were observed for TRA stenosis, which were alleviated through stent implantation. Xiong and colleagues conducted CFD study on an aortorenal bypass case (50). The saphenous vein graft (SVG) bypass had stenosis at the outflow vein tract at follow-up, where asymmetrical flow and high WSS and WSS gradient were observed on CFD analysis. Csonka and colleagues investigated the impact of renal artery-aorta angulation on hemodynamic conditions and its association with renal function (51). An optimal range for the renal artery angle was selected based on relatively constant hemodynamic parameters and higher eGFR values. Overall, CFD offers valuable insights into the hemodynamic changes associated with both RAS and TRA stenosis, which could improve clinical outcomes through more targeted treatments and interventions.

## 5 CFD analysis of aortic lesions involving visceral branches

CFD analysis has been widely employed in the study of aortic lesions to investigate the pathogenesis, predict disease progression and evaluate the therapeutic effects. Those lesions often lead to alterations in the hemodynamic status of the visceral arteries due to the location. However, there is a notable scarcity of CFD studies focusing on the impact of aortic diseases or postoperative conditions on visceral hemodynamics. This section summarizes the major CFD analyses of aortic lesions as well as the prediction of the outcome of different surgical approaches, focusing on their impacts on the involving visceral branches.

### 5.1 Aortic aneurysm

Endovascular aneurysm repair (EVAR) has been developed to treat complex thoracoabdominal aortic aneurysms which require the reconstruction of the visceral branch vessels, especially for patients unfit for open surgery. However, local hemodynamic parameter changes may occur after repair, together with many complications such as stenosis and occlusion, increasing the risk of thrombotic, atherosclerotic, and visceral ischemia. Several endovascular stent graft configurations have been developed to repair aortic aneurysms, which have different impacts on visceral branches. In these CFD studies, the renal arteries are often the focus due to their relatively small diameter, short length, and predominant perpendicular orientation to the aortic axis, making them prone to complications (52).

Sutalo and colleagues developed a single-branch stent-graft model and reported that antegrade branched stent grafts (BSGs) resulted in higher outflow to the renal branch compared to retrograde BSGs for 200-mm conduits (53). Kandail and colleagues conducted a comparative analysis of the hemodynamics of BSGs and fenestrated stent grafts (FSGs) with different renal takeoff angles (ToA) (54). Based on models containing two parallel branch conduits and the iliac bifurcation, they observed larger flow recirculation zones (FRZs) in FSGs, the size of which also highly depended on the renal ToA. They then further examined flared FSGs, finding lower endothelial cell activation potential (ECAP) values at the renal ostia in the geometries with smaller dilation angles, shorter protrusion lengths, and moderate lower limb exercise, which are likely to reduce the risk of thrombosis (55). Suess et al. examined the flow behavior of the above two stent graft configurations for endovascular repair, as well as manifold configuration (56). The results showed near-wall hemodynamic (NWH) parameters with a focus on the renal bridging stent grafts and assessed their ability to maintain branch vessel patency. Georgakarakos and colleagues analyzed the hemodynamic variation resulting from fenestration misalignment, identifying that the values of maximum WSS were influenced by the directions of stented segments (57). Their later study found that the horizontal branch orientation affected the WSS distribution over its length (58). The simulation results of Ou et al. showed higher flow rate and shear stress and smaller FRZs when the renal stent faced caudally, both in custom-made and pivot branch fenestrated endografts (59). These studies above have explored the hemodynamics in target arteries by constructing simplified idealized models. Tricarico and colleagues characterized the anatomic and hemodynamic changes resulting from chimney EVAR (chEVAR) in both the SMA and renal arteries, with analysis of the cross-sectional area, centerline angle, intraluminal pressure, and WSS (60). Their patient-specific models revealed the thresholds to differentiate chimney stent-graft success or occlusion. Negligible hemodynamic differences were found between the chimney configurations with different length of chimney's protrusion (61). Moulakakis and colleagues compared the WSS and flow dynamics for the branch vessels before and after EVAR with fenestrated and chimney techniques. They observed reduced renal perfusion in chimney grafts, probably due to the relatively long curved length (62). Tran et al. found that structural changes in aortic flow geometry after fenestrated EVAR (fEVAR) did not impact estimated renal or visceral branch perfusion metrics or WSS adversely (10). Their further comparative study showed that branched EVAR (bEVAR) caused more subtle reductions in renal perfusion and a larger increase in WSS than fEVAR (63). A recent study by Wang et al. discussed the two stents together and found a better blood flow status in those with a larger tilt angle of the branch stent, smaller branch entry depth, and larger branch stent diameter, especially reflected by their vortex condition (64).Another real-world comparative study between fEVAR and chEVAR presented lower FRZs at parallel grafts' entries but more intense flow regurgitation in visceral stents in chEVAR (65). Those findings provide a CFD method that can aid in evaluating EVAR configurations for patients with aortic aneurysms involving visceral arteries, supporting more personalized treatment decisions and potentially reducing postoperative visceral-artery-related complications.

### 5.2 Aortic dissection

Altered blood flow in patients with complicated aortic dissection (AD) can lead to severe complications such as visceral malperfusion. Jiang et al. made the first attempt to evaluate the therapeutic effect of patients with complicated aortic dissections receiving physician-modified endograft (PMEG) through patient-specific hemodynamic simulations (66). The postoperative models showed increased flow rates and significant reductions of pressure drop and TAWSS in visceral regions, which meant improved blood supply and reduced rupture risk of tears. Due to the absence of patient-specific boundary condition data, they implemented a waveform obtained from literature, which corresponds to normal (healthy) dynamics. This actually overlooked the changes in the complex hemodynamic state after surgery. Lee and colleagues developed a specific-idealized AD model and found a pressure difference between the true lumen and false lumen in axillary cannulation (AC) that would disturb blood flow to the celiac trunk and SMA, while in combined axillary and femoral cannulation (AFC) it showed a slight difference and higher visceral flow (67). The FSI model revealed that a pressure difference can induce clinically significant flap collapse by incorporating flap compliance, and that the stiffness of the intimal flap critically determines the degree of deformation. The simulations produced higher TL velocities and lower FL velocities compared to rigid CFD, indicating that flap deformation redistributes flow in ways that rigid models cannot replicate. Kimura and colleagues simulated the hemodynamics of type B aortic dissection complicated by mesenteric malperfusion. The malperfusion in the coeliac artery and SMA caused by dynamic obstruction was with the treatment of thoracic EVAR, which resulted in true lumen expansion and increased blood flow to the visceral vessels (68). These studies highlight the significant role of CFD analysis in efficacy assessment for AD complicated with visceral malperfusion and may contribute to the improvement and development of the technique.

### 5.3 Other aortic lesions involving visceral branches

Left ventricular assist devices are widely used to treat advanced cardiac heart failure, although this device leads to a high risk for gastrointestinal bleeding via a hemodynamic-mediated mechanism (69). Scardulla and colleagues demonstrated the highest WSS in the left gastric artery and a positive correlation between the celiac trunk angulation and the WSS stress distal to the ostium of the celiac trunk, which may promote bleeding from this vessel (70). Suprarenal abdominal aortic coarctation (SAAC) also alters flow and pressure patterns in visceral regions, particularly affecting renal perfusion. Tossas-Betancourt et al. analyzed the aortorenal blood flow of a SAAC patient to examine the hemodynamic impact of thoracoabdominal bypass (TAB) or patch aortoplasty (PA) (71). Postoperative increases in systolic flow were observed following all surgical interventions, as well as decreases in high-frequency disturbances, aortic pressure, and collateral flow. Nevertheless, only the 0% PA oversizing scenario completely eliminated high-frequency disturbances. These studies suggest the potential of CFD in the study of aortic lesions involving visceral arteries and help advance treatment strategies.

## 6 Challenges and future directions

Existing studies have demonstrated that CFD analysis is a useful tool for understanding the complex hemodynamic interactions in lesions of or involving the visceral arteries. However, several challenges and limitations remain, particularly concerning the accuracy, clinical applicability, and generalizability of CFD-based findings.

One major challenge is the simplification of models and computational assumptions. Due to the complex and highly variable anatomy of patient-specific visceral arteries, many studies rely on idealized geometries to reduce computational load (53, 54, 56–59).

Mesh sensitivity analysisis considered a best practice in engineering simulations, which systematically refining the mesh until key output variables converge. However, a survey of the literature reviewed here reveals that many visceral artery CFD studies do not report such convergence tests. Given the computational cost of highly refined meshes in complex branching geometries, a trade-off exists between accuracy and feasibility. We recommend that future studies explicitly document mesh independence and report metrics like Grid Convergence Index (GCI) or explicitly detail near-wall prism layer resolution (y^+^ values), enabling readers to gauge the reliability of the results. GCI method was proposed by Roache to calculate the discretization error using three systematically refined meshes (coarse, medium, fine), and a GCI below 5% is generally considered acceptable (72). A mesh independence analysis of aortic flow simulations clearly demonstrated that appropriate grid resolution significantly influences the accuracy and reliability of WSS results (73). It must be noted that parameters such as TAWSS and OSI are highly sensitive to boundary layer resolution and without verifiable convergence, these clinical markers remain speculative. Near-wall resolution should be expressed as y⁺ values for WSS-related parameters, with a recommended target of y⁺ < 1 for laminar or transitional flow simulations, or y⁺ ≈ 30–300 with wall functions for turbulent flow. As for element type, hexahedral meshes were considered to offer the highest accuracy and efficiency. The flow-adaptive polyhedral mesh has shown with shorter solution convergence time compared to hexahedral meshes and better results compared to conventional adaptive and nonadaptive tetrahedral meshes (73).

Vessel walls are often assumed to be rigid and nondeformable, while blood is treated as an incompressible Newtonian fluid, despite its inherently non-Newtonian behavior. These simplifications facilitate simulations but can compromise the accuracy and physiological relevance of results (74). Rigid walls cannot store energy during systole via elastic expansion nor can it release energy during diastole via recoil. Therefore, they artificially inflate systolic pressure gradients and exaggerate wave reflections at arterial junctions and truncated outlets. A concrete understanding of this bias is important for clinicians. Improving FSI techniques and leveraging high-performance computing resources will enable more accurate and detailed hemodynamic assessments. Streamline analysis of Kimura et al. (68) suggested that visceral perfusion was repeatedly disrupted by intermittent covering of vessel orifices by the intimal flap. However, because the model assumed a rigid intimal flap, the actual dynamic motion of the flap and its causal relationship with pressure differences could not be quantified or mechanistically explained. Rigid-wall models may underestimate the risk of dynamic malperfusion because they cannot capture the progressive collapse of the TL under pressure overload. In contrast, Lee et al. (67) performed an FSI simulation using a specific-idealized aortic dissection model with a deformable intimal flap. They directly compared two cannulation strategie sand quantified the pressure difference between the TL and FL. Future studies should aim to integrate patient-specific flap properties and validate FSI predictions against *in vivo* measurements such as 4D flow MRI or invasive pressure monitoring. On the other hand, FSI are computationally intensive and time-consuming, which restricts their application to small sample cohorts and impairs the generalizability of findings. Future studies may benefit from the integration of biaxial biomechanical testing to enhance the accuracy of vascular wall behavior modeling (24).

For clinicians, a tangible understanding of the bias introduced by rigid-wall assumptions is important. In pulsatile flow systems, rigid walls artificially inflate pressure gradients because they cannot store energy during systole (via vessel expansion) and release it during diastole (via elastic recoil). This results in overestimation of systolic pressure peaks and underestimation of diastolic pressure maintenance. Furthermore, rigid walls exaggerate wave reflection because the natural damping and phase-shifting effects of compliant vessels are absent, leading to spurious oscillatory patterns in derived parameters such as OSI. Therefore, while rigid-wall simulations may be acceptable for comparing relative hemodynamic changes (e.g., pre- vs. post-stent) under identical assumptions, absolute values—especially pressure-related metrics—should be interpreted with caution.

Additionally, in EVAR configuration studies, the influence of strut texture and its interaction with the aortic wall should be incorporated to better replicate post-implantation hemodynamics. Another critical gap is the inadequate modeling of endothelialization and thrombosis following stent placement, processes that significantly impact long-term graft patency and patient outcomes.

The lack of comprehensive clinical data further limits the accuracy and translational potential of CFD studies. Visceral arteries exhibit highly complex and individualized anatomical variations, making it challenging to acquire sufficient clinical data that accurately captures their geometry and hemodynamic conditions. Many CFD studies rely on retrospective datasets, where patient-specific hemodynamic parameters such as inlet and outlet pressures or flow rates are unavailable. Consequently, generalized relationships between body weight and cardiac output (75) or literature-derived boundary conditions are often applied, which may not accurately reflect individual patient physiology. Some studies applied the same boundary conditions in preoperative and postoperative models, which also makes it difficult to realistically simulate the effects of complex procedures.

While most current CFD studies impose simplified boundary conditions such as fixed flow splits or generic Windkessel parameters, the visceral arteries are unique in that they perfuse highly autoregulated organs. Multi-scale models (MSM) address this limitation by coupling a detailed 3D CFD model of the target vessel with 0D lumped parameter models that represent the distal vasculature and organ beds. This approach allows the boundary conditions to evolve dynamically based on the computed pressure and flow from the 3D domain, thereby enabling more physiologically realistic simulations of conditions such as renal artery stenosis or mesenteric ischemia. By limiting computationally intensive 3D simulations to regions of interest and using 0D models for the rest of the system, MSM significantly reduces simulation time and resource requirements. However, the standard three-element Windkessel model assumes fixed or linear distal resistances and cannot replicate the dynamic autoregulation of renal and mesenteric beds. In the kidney, pressure flow autoregulation maintains stable perfusion over a wide pressure range. A constant-resistance model can mispredict flow changes caused by pressure, potentially overestimating the ischemic burden in renal artery stenosis. In the mesenteric circulation, standard models cannot simulate the marked postprandial hyperemic response nor exerciseinduced flow redistribution. Furthermore, conventional outflow conditions often produce artificial wave reflections at truncated outlets that distort pressure and flow waveforms in visceral branches. More advanced impedance boundary conditions mitigate these reflections by modeling the frequency-dependent behavior of the distal vasculature.

Additionally, parameters such as TAWSS cannot be directly obtained with current clinical imaging modalities, making experimental validation of CFD findings difficult. Consequently, researchers can only validate velocity fields and flow rates, while the accuracy of parameters like WSS depends on the correctness of all model assumptions. The most common form of validation is qualitative or semi-quantitative comparison based on imaging. Researchers visually compare simulated streamlines, velocity fields, or pressure distributions with clinical images such as 4D-Flow MRI to directly acquire patient-specific velocity fields and compared them with CFD simulation results, validating the ability of the models to capture complex flow patterns (20). However, this type of validation is typically qualitative and difficult to quantify errors. When patient-specific data are unavailable, some studies have used *in vitro* models or standard waveforms from the literature for indirect validation (53, 54). Nevertheless, this approach cannot account for individual patient variability. Some studies have indirectly validated the predictive value of their models by retrospectively correlating CFD-derived parameters with patient clinical prognoses. For instance, Tricarico et al. (60) found that patients who developed stent-graft occlusion within one month of chimney EVAR had significantly higher simulated systolic pressure gradients and WSS than those who did not, thereby establishing an association between hemodynamic parameters and clinical events. Park et al. (21) also compared simulated high-stress regions with the location of lesions in SISMAD, validating the role of mechanical factors in disease pathogenesis. This kind of retrospective validation cannot exclude the interference of other confounding factors. To enhance the clinical credibility of CFD models, future research should prioritize the integration of real-time blood flow data from Doppler ultrasound, dynamic digital subtraction angiography (DSA), or four-dimensional flow MRI to enhance boundary condition accuracy and improve clinical translation.

Advancements in computational power and artificial intelligence present exciting opportunities for the future of CFD applications in visceral artery research. Large-scale, patient-specific studies incorporating real-world data will be essential for improving the reliability and clinical relevance of CFD simulations. The development of machine learning algorithms for automated image segmentation can significantly streamline model generation (76), particularly in the context of the intricate and highly variable anatomical structures of visceral arteries. Machine learning algorithms also have been proposed to predict the hemodynamic environment, in which the physics-informed neural network (PINN) can quickly and highly resolve the flow information hidden behind the concentration field through the distribution of the substance concentration in the blood vessels. It shows particular potential in overcoming the inability to directly measure critical variables such as pressure fields and wall shear stress. PINNs are trained not only on sparse measurement data but also on the governing Navier–Stokes equations as physical constraints. This capability enables inverse problem solving, whereby low-resolution, noisy velocity data from routine clinical imaging modalities include 4D-flow MRI or DSA can be used to reconstruct high-fidelity, continuous velocity and pressure fields that are not directly measurable. Raissi et al. successfully simulated blood flow in a local model of an intracranial aneurysm (77), and Buoso et al. applied PINN to personalized left ventricular biomechanical simulation (78), but no analytical process or results have been reported for large-scale clinical DSA data in lesions of or involving the visceral arteries. For visceral artery applications, it could address limitations like the lack of patient-specific boundary condition data and the difficulty of validating WSS predictions that noted earlier in this section. By assimilating sparse *in vivo* measurements, PINNs can infer physiologically consistent inflow and outflow conditions and estimate pressure gradients without invasive catheterization.

Addressing these challenges requires a multidisciplinary approach. Close collaboration among vascular surgeons, radiologists, and biomedical engineers is crucial to refining computational models, integrating advanced imaging techniques, and ensuring that CFD findings are clinically meaningful. Additionally, the involvement of specialists in specific visceral organ fields, such as liver surgeons, is essential. These experts can help raise clinically relevant questions, provide insights into disease-specific hemodynamics, and determine whether CFD could be effectively applied to solve pressing clinical problems. By fostering interdisciplinary discussions and leveraging expertise across multiple specialties, CFD research can be further tailored to address real-world clinical needs and ultimately leading to improved patient outcomes.

## 7 Conclusion

In conclusion, CFD is a powerful tool for analyzing the hemodynamics and aiding in disease assessment, treatment planning, as well prognosis prediction for lesions of or involving the visceral arteries. For primary visceral artery diseases such as mesenteric stenosis, renal artery stenosis, and visceral aneurysms, CFD has provided valuable insights into local hemodynamic mechanisms and supported surgical planning. For aortic pathologies with visceral branch involvement, including complex aneurysms and dissections, CFD makes it possible to evaluate different endovascular repair configurations and their impact on branch vascular perfusion and long-term patency. However, challenges such as model simplifications, computational constraints, and insufficient clinical data limit its accuracy and applicability, keeping it still in its early stages. The path to clinical translation requires further engineering advances and multidisciplinary collaboration. A multidisciplinary approach involving vascular surgeons, radiologists, engineers, and organ-specific specialists is essential to translating CFD insights into improved patient care. Future advancements in fluid-structure interaction modeling, real-time hemodynamic data integration, and machine learning will enhance CFD's clinical utility.

## Appendix A

((computational modeling) OR (computational fluid dynamics) OR (computational solid mechanics) OR (fluid-structure interaction) OR (finite element method) OR (fluid element analysis)) AND ((aneurysm) OR (dissection) OR (Stenosis) OR (Occlusion) OR (Rupture) OR (Thrombosis) OR (fenestration) OR (branch) OR (chimney)) AND ((visceral) OR (spleen) OR (renal) OR (mesenteric) OR (celiac)).
