# Supplementary material for: Computational studies on visceral artery lesions and aortic pathologies involving visceral branches: a comprehensive review for the clinician
Source: Front Cardiovasc Med. 2026 Jul 10;13:1856457. doi: 10.3389/fcvm.2026.1856457 (PMC13395936; doi:10.3389/fcvm.2026.1856457)
Supplement: Supplementary file 1 [file Table1.docx]

**S1. Quality checklist for CFD/FSI studies**

| Item | Criteria | Score (0/1/2) |
| --- | --- | --- |
| 1 | Geometry source and segmentation (0=idealized, 1=patient‑specific single phase, 2=patient‑specific with segmentation reported) |  |
| 2 | Mesh convergence analysis (0=not reported, 1=reported but incomplete, 2=systematic with GCI or ≥3 refinements) |  |
| 3 | Boundary conditions (0=constant/idealized, 1=literature waveform or single measurement, 2=patient‑specific with Windkessel/impedance) |  |
| 4 | Flow regime justification (0=not stated, 1=stated without Reynolds number or sensitivity, 2=explicitly justified with Re or comparison) |  |
| 5 | Validation (0=none, 1=qualitative or comparison with imaging, 2=quantitative against invasive data, 4D flow, or clinical outcomes) |  |
| 6 | Clinical relevance and reproducibility (0=general parameters only, 1=potentially clinically meaningful, 2=directly linked to decision/outcome) |  |

**S2. Quality assessment scores of selected studies**

| Study (first author, year) | Geometry | Mesh convergence | Boundary conditions | Turbulence | Validation | Clinical relevance | Total (0–12) | Quality |
| --- | --- | --- | --- | --- | --- | --- | --- | --- |
| Šutalo et al. 2008 | Idealized | Yes | Literature flow split | k‑ε | Experimental | Yes | 9 | High |
| Kagadis et al. 2008 | Patient‑specific | Yes (6 meshes) | Sinusoidal pressure | SST (Re~1100) | Literature + ultrasound | Yes | 11 | High |
| Park et al. 2011 | Idealized | No | Literature pressure | Not stated | Qualitative (lesion site) | Yes | 4 | Low |
| Childress et al. 2014 | Idealized | Yes | Inlet waveform + Windkessel | Non‑Newtonian (Carreau) | In vitro (Kung model) | Yes (tumor targeting) | 9 | High |
| Kandail et al. 2015 | Idealized | Yes | Literature + 3‑EWM | Non‑Newtonian (Quemada) | None | Yes | 7 | Moderate |
| Kandail et al. 2016 | Idealized | Yes | Literature + 3‑EWM | Newtonian (laminar) | None | Yes | 7 | Moderate |
| Suess et al. 2016 | Idealized | Partially | Flow split outlets | Low Re turbulence (induced) | None | Yes | 5 | Low |
| Georgakarakos et al. 2016 | Idealized | No | Literature + pressure outlets | Non‑Newtonian (Carreau‑Yasuda) | None | Yes | 5 | Low |
| Ou et al. 2017 | Idealized (patient‑based) | No | Literature + pressure outlets | Newtonian (laminar) | None | Yes | 4 | Low |
| Tricarico et al. 2017 | Patient‑specific (6 chEVAR) | Yes (ΔWSS<10%) | Scaled literature + flow split | Laminar (Re 230–590) | Retrospective clinical outcomes | Yes | 8 | Moderate |
| Scardulla et al. 2017 | Patient‑specific (5 LVAD) | No | Steady (LVAD constant) + in vitro | Laminar | In vitro (3D‑printed + US) | Yes | 7 | Moderate |
| Yuhn et al. 2019 | Patient‑specific (1D‑0D) | Indirect (TAWSS steady) | 1D‑0D Windkessel | N/A (1D) | CTA morphology | Yes | 7 | Moderate |
| Moulakakis et al. 2019 | Patient‑specific (2 patients) | Yes (1.7M elements) | Literature + pressure outlets | Laminar (Re<4000) | None | Yes | 6 | Moderate |
| Tossas‑Betancourt et al. 2020 | Patient‑specific (1 child) | No | PC‑MRI calibrated + Windkessel | Laminar (FSI) | Clinical data (PC‑MRI, pressure) | Yes | 7 | Moderate |
| Du et al. 2021 | Patient‑specific | No | Velocity inlet + resistance outlets | k‑ε (not justified) | Group comparison | Partial | 5 | Low |
| Tran et al. 2021 (fEVAR) | Patient‑specific (10 fEVAR) | Yes (<1% variance) | Allometrically scaled + 3‑EWM | Newtonian (laminar) | None (paired pre/post) | Yes | 8 | Moderate |
| Gao et al. 2022 | Patient‑specific (4D‑CTA) | Yes (multiple phases, SD<2%) | ECG‑gated fitted curves | k‑ε (standard) | Multi‑phase comparison | Yes | 10 | High |
| Jia et al. 2022 | Patient‑specific | Yes (grid sensitivity) | Patient‑specific + FSI | k‑ω SST (transition) | Histology + lesion site | Yes | 9 | High |
| Li et al. 2022 (VAA) | Patient‑specific | Yes (2M elements, Δ<1%) | Literature | Laminar (not stated) | Aneurysm vs para‑aneurysm | Partial | 5 | Low |
| Mei et al. 2023 | Patient‑specific | No | Patient‑specific peak velocity | k‑ω SST (transition) | Qualitative (SMAS vs SMAD) | Yes | 8 | Moderate |
| Zhao et al. 2023 | Patient‑based idealized | Partially | Literature + pressure outlet | Laminar (not stated) | Qualitative parametric | Yes | 7 | Moderate |
| Csonka et al. 2023 | Idealized (angle variation) | No | Literature velocity waveform | Realizable k‑ε (separation) | eGFR correlation | Yes | 7 | Moderate |
| Jiang et al. 2023 | Patient‑specific (6 PMEG) | Yes (mesh sensitivity) | Literature + 3‑EWM | Laminar | Clinical 1‑year outcomes | Yes | 8 | Moderate |
| Lee et al. 2023 | Idealized (specific‑idealized AD) | Yes (1.6M fluid, 55k solid) | 4D flow MRI‑based + FSI | k‑ω SST | In vitro 4D flow MRI | Yes | 9 | High |
| Brand et al. 2024 | Idealized (chEVAR) | Yes (2M elements, Δ<1%) | Pulsatile + constant flow split | Newtonian + Carreau | None | Yes | 7 | Moderate |
| Malatos et al. 2024 | Patient‑specific (3 fEVAR, 3 chEVAR) | Yes (<2% change) | Multiscale (1D‑0D) | Newtonian (laminar) | None | Yes | 7 | Moderate |
| Kimura et al. 2024 | Patient‑specific (1 TEVAR) | No | Literature + pressure outlets | Not stated (laminar assumed) | Qualitative pre/post | Yes | 6 | Moderate |
| Tran et al. 2024 (fEVAR vs bEVAR) | Patient‑specific (10 fEVAR, 10 bEVAR) | Yes (0.5‑1.0‑0.3 mm) | Allometrically scaled + 3‑EWM | Newtonian (laminar) | None | Yes | 8 | Moderate |
| Tatari et al. 2024 | Patient‑specific | Yes (4.2M vs 8.98M, Δ<2%) | Literature + resistance outlets | Laminar (not stated) | With vs without collaterals | Yes | 8 | Moderate |
| Zhang et al. 2024 | Patient‑specific (62 SISMAD) | Partially | Literature waveform | Not stated | Qualitative (clinical types) | Yes | 8 | Moderate |
| Wang et al. 2025 | Patient‑specific (6 cases) | No | Constant velocity + constant pressure | Newtonian (laminar) | Clinical occlusion correlation | Yes | 6 | Moderate |
| Soliveri et al. 2025 | Patient‑specific | Yes (3 meshes, Δ<1%) | Patient‑specific peak velocity + calibrated outlets | Laminar (steady) | Clinical decision (AUC=0.988) | Yes | 10 | High |
| Wei et al. 2025 | Patient‑specific | Yes (172k elements) | Literature inlet, constant outlet | Laminar (Re~1800) | Qualitative (TL vs FL) | Partial | 6 | Moderate |
| Xu et al. 2025 | Patient‑specific (70 SISMAD) | No | Fixed flow, outflow | Steady laminar | Clinical outcome groups | Yes | 6 | Moderate |

Quality: High (≥9), Moderate (6–8), Low (≤5)
